# The heterogeneity of symptom reporting across study sites: a secondary analysis of a randomised placebo-controlled multicentre antimalarial trial

**DOI:** 10.1186/s12874-023-02022-3

**Published:** 2023-09-04

**Authors:** Kamala Thriemer, Robert James Commons, Megha Rajasekhar, Tamiru Shibiru Degaga, Krisin Chand, Nguyen Hoang Chau, Ashenafi Assefa, Mohammad Nader Naddim, Ayodhia Pitaloka Pasaribu, Awab Ghulam Rahim, Inge Sutanto, Tran Tinh Hien, Asrat Hailu, Mohammad Anwar Hasanzai, Lenny L. Ekawati, Adugna Woyessa, Tedla Teferi, Naomi Waithira, Walter R. J. Taylor, Benedikt Ley, Arjen Dondorp, J. Kevin Baird, Nicholas J. White, Nicholas P. Day, Ric N. Price, Julie A. Simpson, Lorenz von Seidlein

**Affiliations:** 1 Global and Tropical Health Division, Menzies School of Health Research and Charles Darwin University, Darwin, Australia; 2 Medical Services, Grampians Health Ballarat, Ballarat, Australia; 3 Centre for Epidemiology and Biostatistics, Melbourne School of Population and Global Health, University of Melbourne, Melbourne, VIC Australia; 4 College of Medicine & Health Sciences, Arbaminch University, Arbaminch, Ethiopia; 5 Oxford University Clinical Research Unit, Jakarta, Indonesia; 6 Faculty of Medicine, Universitas Indonesia, Jakarta, Indonesia; 7 Oxford University Research Unit, Hospital for Tropical Diseases, Ho Chi Minh City, Vietnam; 8 Ethiopian Public Health Institute, Addis Ababa, Ethiopia; 9 Institute for Global Health and Infectious Disease, Chapel Hill, NC USA; 10 Health Protection and Research Organisation, Kabul, Afghanistan; 11 Universitas Sumatera Utara, Medan, Indonesia; 12 Nangarhar Medical Faculty, Ministry of Higher Education, Nangarhar University, Jalalabad, Afghanistan; 13 College of Health Sciences, Addis Ababa University, Addis Ababa, Ethiopia; 14 Arba Minch General Hospital, Arba Minch, Ethiopia; 15 Mahidol Oxford Tropical Medicine Research Unit, Bangkok, Thailand; 16 Centre for Tropical Medicine, Nuffield Department of Medicine, University of Oxford, Oxford, UK

**Keywords:** Clinical trial, Safety, Tolerability, Symptom reporting, Trial design

## Abstract

**Introduction:**

Symptoms reported following the administration of investigational drugs play an important role in decisions for registration and treatment guidelines. However, symptoms are subjective, and interview methods to quantify them are difficult to standardise. We explored differences in symptom reporting across study sites of a multicentre antimalarial trial, with the aim of informing trial design and the interpretation of safety and tolerability data.

**Methods:**

Data were derived from the IMPROV trial, a randomised, placebo-controlled double blinded trial of high dose primaquine to prevent *Plasmodium vivax* recurrence conducted in eight study sites in Afghanistan, Ethiopia, Indonesia and Vietnam. At each follow up visit a 13-point symptom questionnaire was completed. The number and percentage of patients with clinically relevant symptoms following the administration of primaquine or placebo, were reported by study site including vomiting, diarrhoea, anorexia, nausea, abdominal pain and dizziness. Multivariable logistic regression was used to estimate the confounder-adjusted site-specific proportion of each symptom.

**Results:**

A total of 2,336 patients were included. The greatest variation between sites in the proportion of patients reporting symptoms was for anorexia between day 0 and day 13: 97.3% (361/371) of patients in Arba Minch, Ethiopia, reported the symptom compared with 4.7% (5/106) of patients in Krong Pa, Vietnam. Differences attenuated slightly after adjusting for treatment arm, age, sex, day 0 parasite density and fever; with the adjusted proportion for anorexia ranging from 4.8% to 97.0%. Differences between sites were greater for symptoms graded as mild or moderate compared to those rated as severe. Differences in symptom reporting were greater between study sites than between treatment arms within the same study site.

**Conclusion:**

Despite standardised training, there was large variation in symptom reporting across trial sites. The reporting of severe symptoms was less skewed compared to mild and moderate symptoms, which are likely to be more subjective. Trialists should clearly distinguish between safety and tolerability outcomes. Differences between trial arms were much less variable across sites, suggesting that the relative difference in reported symptoms between intervention and control group is more relevant than absolute numbers and should be reported when possible.

**Trial registration:**

Clinicaltrials.gov: NCT01814683; March 20^th^, 2013.

**Supplementary Information:**

The online version contains supplementary material available at 10.1186/s12874-023-02022-3.

## Background

Clinical trials are designed to evaluate drug safety and efficacy. The assessment of safety usually involves identifying adverse events (AEs), that can be detected either by laboratory testing, clinical assessment or patient questioning for symptoms. While laboratory tests can be standardised, patient questioning methods are more complex and difficult to standardise. Placebo controlled double-blinded designs are important to ensure questioning is independent of treatment allocation. Patient symptoms questioning relies on individual patient reporting and information that can rarely be corroborated through other means. Investigators usually grade symptoms reported by the trial participant on a scale from mild, through moderate, to severe; however, grading matrices are variable between different studies and often specifically defined within a study protocol. Subjective assessments rely on the perceptions of the trial participants and the same question may trigger a range of responses in different individuals [1, 2]. For instance, a sensation of discomfort may be ignored or considered as mild by one study participant, while perceived as moderate to severe requiring medication by another. The wording of interview questions and the attitude of the interviewer can also influence the trial participants’ responses [3–5]. A systematic Cochrane review comparing different methods used within clinical drug trials to elicit information about AEs showed that detailed questioning of study subjects resulted in more events being reported compared to a general interview. This was particularly true for mild symptoms, while more severe AEs were also reported in the open enquiry [6].

The ‘improving the radical cure of vivax malaria’ (IMPROV) trial (NCT01814683) compared the efficacy, safety, and tolerability of two primaquine regimens for the radical cure of vivax malaria in 8 sites across 4 countries [7]. The study was randomised, double-blind, and placebo-controlled, since the use of hypnozoiticidal drugs like primaquine for the radical cure of vivax malaria was, at the time, not the standard of care in most of the study sites [8]. The safety and tolerability assessment consisted of two components. First, laboratory evaluations comprised haemoglobin measurement and urine colour assessment, which are critical in the safety evaluation of primaquine which can cause haemolysis. Second, a symptom checklist was used to assess vomiting, diarrhoea, anorexia, nausea, abdominal pain, dizziness and other symptoms [9].

In this analysis, we aimed to explore potential differences in symptom reporting across study sites and whether study site variation could be adjusted for, to inform future clinical trial designs and the interpretation of safety and tolerability data.

## Methods

### Study design

Data used in this analysis were derived from the IMPROV trial. The design of the trial [10] as well as main results have been published previously [7]. In brief, patients presenting with uncomplicated vivax malaria, who had fever or a history of fever within the previous 48 h, were aged older than 6 months, weighed at least 5 kg, had a haemoglobin concentration of ≥ 9 g/dL, and had normal glucose-6-phosphate-dehydrogenase (G6PD) status, as assessed by the fluorescent spot test, were eligible for enrolment. Exclusion criteria were pregnancy or lactation, known previous haemolytic episodes or blood transfusion in the past 90 days, signs of severe malaria or inability to tolerate oral treatment, known hypersensitivity to study drugs, or concomitant medication with the potential to cause haemolysis or interfere with the pharmacokinetics of the study drugs. Eligible patients were enrolled into the study and treated with schizontocidal treatment plus either high dose primaquine (7 mg/kg total dose) over 7 days or over 14 days or received placebo. The placebo had an identical appearance to primaquine but was made of a biologically inactive agent. Participants were requested to return for follow up visits daily from day 0 through to 13, then weekly from week 3 through to 8 and then monthly from months 3 through to 12 [10].

### Study sites

This study included patients recruited in eight study sites across four countries: Afghanistan, Ethiopia, Indonesia and Vietnam [7]. The two sites located in Afghanistan and in Vietnam were relatively close to each other in distance and managed by the same country investigator and study teams. The two sites in Ethiopia and Indonesia respectively were geographically further apart and managed by different teams. Previous clinical trial experience ranged from extensive to very limited experience. All sites received standardised training before the start of the study including detailed training how to complete the case record form (CRF).

### Data collection of symptoms

At each follow up visit a symptom questionnaire was completed. Patients were encouraged to report to the study centre if they became unwell between scheduled visits. The symptom questionnaire consisted of a 13-point symptom checklist for fever/hot body, headache, muscle/joint aches and pain, abdominal pain, poor appetite, nausea, vomiting, diarrhoea, passing red/brown/black urine, skin rash, dizziness, shortness of breath and itching. The questionnaire was available in English at all sites, except for the Indonesian sites where it was translated into Bahasa. Each symptom could be either present at the time of the interview or reported as occurring since the last visit and was graded according to study specific standard operating procedures (SOP) as either mild, moderate, severe or potentially life threating using definitions outlined in Table 1 [10]. Signs identified during a medical examination were not included in the checklist.

**Table 1:** Severity grading scale for symptom reporting

Systemic (General)	Mild (Grade 1)	Moderate (Grade 2)	Severe (Grade 3)	Potentially life threatening (Grade 4)
Nausea/ vomiting	No interference with activity or 1 – 2 episodes/24 h	Some interference with activity or > 2 episodes/24 h	Prevents daily activity, requires outpatient IV hydration	Emergency department visit or hospitalisation for hypotensive shock
Diarrhoea	2 – 3 loose stools or < 400 g/24 h	4 – 5 stools or 400 – 800 g/24 hours	6 or more watery stools or > 800 g/24 h or requires outpatient IV hydration	Emergency department visit or hospitalisation
Other illness e.g. anorexia, abdominal pain, dizziness	No interference with activity	Some interference with activity not requiring medical intervention	Prevents daily activity and requires medical intervention	Emergency department visit or hospitalisation

### Data analysis

The number and proportion of patients who had the most clinically relevant symptoms (vomiting, diarrhoea, anorexia, nausea, abdominal pain and dizziness) following the administration of primaquine or placebo are presented by study site and treatment. The confounder-adjusted proportion of the presence of each symptom at least once between day 0 and 13 for each study site was estimated following multivariable logistic regression with each confounder (age, sex, day 0 parasitaemia, treatment arm, and presence of fever on day 0) set at the population mean/prevalence value of the overall trial. Schizontocidal treatment was not adjusted for due to collinearity with study site (chloroquine for all patients in Ethiopia, Vietnam and Afghanistan, dihydroartemisinin-piperaquine for patients in Indonesia). A sensitivity analysis was performed excluding patients less than 5 years, whose parents or guardians may have reported their symptoms.

To explore whether the observed variation between study sites may be due to differences in acute malaria symptoms the analyses were repeated for each symptom restricted to the time after acute malaria symptoms are expected to subside (days 3 to 13). To further explore whether the reported presence or absence of symptoms in an individual on day 0 or 1 could explain the variation between study sites, the models were repeated including symptom presence on day 0 or 1 as a covariate.

To investigate whether variation in symptom reporting was impacted by severity, the presence of severe symptoms (grade 3) was compared between study sites. The confounder-adjusted proportion of symptoms between day 3 and 13 was described for each treatment arm using the previous multivariable logistic regression model and compared across sites to explore within and between-study site variation in symptoms.

All statistical analyses were performed using Stata version v17.0 (StataCorp, US). Confounder-adjusted proportions of symptoms were estimated using the margins command.

## Results

**Fig. 1:**
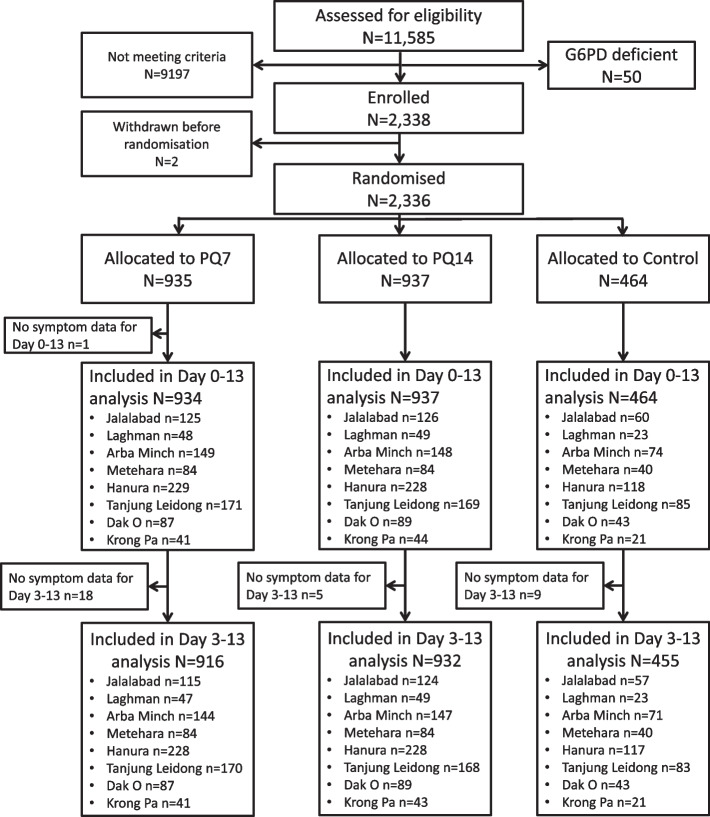
CONSORT chart of patients included in the study

In total, 2,336 patients were randomised into the study [7]. Treatment arms were equally divided across study sites. The number of study participants at each site varied between 106 (4.5%) in Krong Pa, Vietnam and 575 (24.6%) in Hanura, Indonesia. Data from 2,335 patients were available for symptom assessment between day 0 and day 13, and 2,303 for assessment between day 3 and 13 (Fig. 1). The median age of patients was 16 years (interquartile range (IQR) 10 to 26 years), with a total of 154 (6.6%) under the age of 5 years, 63% (1,467/2,336) of participants were male, and their median weight was 47kg (IQR 26 to 57 kg) (Table 2).

**Table 2:** Baseline characteristics by study site

Country	Site	Patients	Age (years)	Patients under 5 years	Male sex	Weight (kg)	Baseline parasitaemia^a^ (/μl)	Baseline presence of fever
		**n**	**Median (IQR)**	**N (%)**	**N (%)**	**Median (IQR)**	**Median (IQR)**	**N (%)**
Afghanistan	Jalalabad	311	14 (9.0—22.0)	23 (7.4%)	234 (75%)	41.0 (22.5—61.3)	1064.8 (666.7–2296.3)	89 (28.6%)
	Laghman	120	11 (7.5—16.0)	6 (5.0)	71 (59%)	31.9 (21.8—46.9)	2194.4 (611.1–5750.0)	20 (16.7%)
Ethiopia	Arba Minch	371	16 (10.0—20.0)	43 (11.6%)	195 (53%)	47.0 (24.0—58.0)	12,500.0 (3037.0–52500.0)	1 (0.3%)
	Metehara	209	16 11.0—27.0)	11 (5.3%)	136 (65%)	47.1 (29.0—55.5)	883.2 (314.4–21,250.0)	45 (21.5%)
Indonesia	Hanura	575	14 (8.0—27.0)	51 (8.9%)	300 (52%)	38.0 (21.3—52.0)	1674.1 (440.7–4633.3)	14 (2.4%)
	Tanjung Leidong	425	17 (11.0—30.0)	19 (4.5%)	251 (59%)	49.0 (27.4—58.1)	7133.3 (4466.7–11,364.8)	100 (23.5%)
Vietnam	Dak O & Bu Gia Map	219	22 (16.0—32.0)	1 (0.5%)	184 (84%)	52.0 (45.0—60.0)	10,000.0 (4565.9–17,500.0)	13 (5.9%)
	Krong Pa	106	25 (22.0—30.0)	0 (0%)	96 (91%)	55.0 (52.0—60.0)	10,000.0 (2351.9–25,000.0)	3 (2.8%)
**Total**		**2,336**	**16 (10.0—26.0)**	**154 (6.6%)**	**1467 (63%)**	**46.6 (26.0—57.0)**	**3985.2 (963.0–12037.0)**	**285 (12.2%)**

^a^Baseline parasitaemia data were missing for 48 patients

### Variation across study sites

The widest range of the proportion of patients reporting a specific symptom across the sites was for anorexia. A total of 97.3% (361/371) of patients in Arba Minch, Ethiopia reported anorexia between day 0 and day 13 compared with 4.7% (5/106) of patients in Krong Pa, Vietnam. The proportion of patients at the remaining sites reporting anorexia fell between these maximum and minimum values (Table 3). The differences between the lowest and highest percentage of other symptoms across the sites were less extreme: percentages ranged from 6.6% to 83.3% for abdominal pain, 0% to 75.2% for dizziness, 3.8% to 85.9% for nausea, 3.8% to 50.3% for vomiting, and 1.9% to 24.0% for diarrhoea.

**Table 3:** Participants reporting symptoms at least once between day 0 and day 13 by study site

Country	Site	Patients	Vomiting	Diarrhoea	Anorexia	Nausea	Abdominal pain	Dizziness
			**n (%)**	**n (%)**	**n (%)**	**n (%)**	**n (%)**	**n (%)**
Afghanistan	Jalalabad	311	37 (11.9%)	15 (4.8%)	99 (31.8%)	73 (23.5%)	62 (19.9%)	47 (15.1%)
	Laghman	120	11 (9.2%)	5 (4.2%)	47 (39.2%)	27 (22.5%)	11 (9.2%)	1 (0.8%)
Ethiopia	Arba Minch	371	200 (53.9%)	89 (24.0%)	361 (97.3%)	284 (76.5%)	235 (63.3%)	279 (75.2%)
	Metehara	208	51 (24.5%)	11 (5.3%)	109 (52.4%)	96 (46.2%)	36 (17.3%)	4 (1.9%)
Indonesia	Hanura	575	289 (50.3%)	53 (9.2%)	407 (70.8%)	426 (74.1%)	334 (58.1%)	194 (33.7%)
	Tanjung Leidong	425	212 (49.9%)	15 (3.5%)	378 (88.9%)	365 (85.9%)	354 (83.3%)	112 (26.4%)
Vietnam	Dak O & Bu Gia Map	219	20 (9.1%)	15 (6.8%)	15 (6.8%)	9 (4.1%)	33 (15.1%)	4 (1.8%)
	Krong Pa	106	4 (3.8%)	2 (1.9%)	5 (4.7%)	4 (3.8%)	7 (6.6%)	0 (0%)
**Total**		**2,335**	**824 (35.3%)**	**205 (8.8%)**	**1,421 (60.9%)**	**1,284 (55.0%)**	**1,072 (45.9%)**	**641 (27.5%)**

**Fig. 2:**
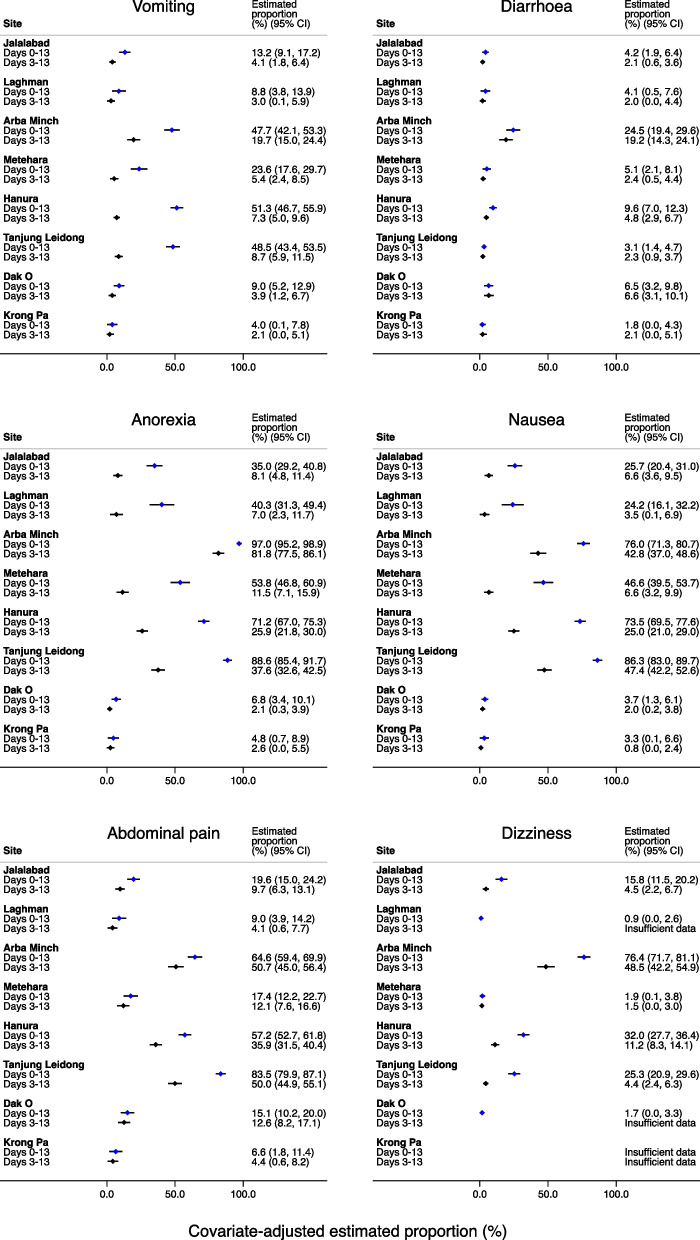
Covariate-adjusted estimate (95% CI) of proportion of patients reporting symptoms between day 0 and 13 and day 3 and 13 Legend: Covariate-adjusted site-specific estimated proportions were generated from logistic regression models adjusting for treatment arm, age, sex, day 0 parasite density and day 0 fever, with all covariates set at mean/prevalence values for all trial patients

**Fig. 3:**
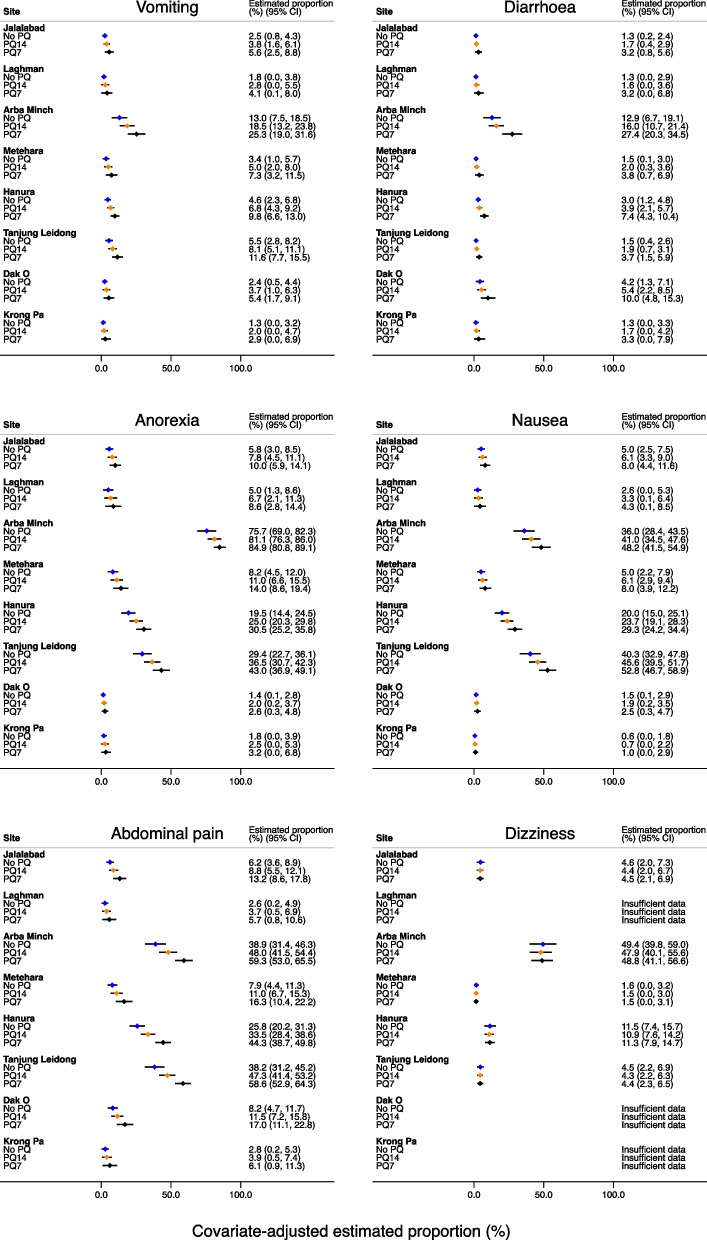
Covariate-adjusted estimate (95% CI) of proportion of patients reporting symptoms at least once between day 3 and day 13 by treatment arm Legend: PQ – primaquine; PQ14 – 14-day course of primaquine; PQ7 – 7-day course of primaquine; Covariate-adjusted site-specific estimated proportions for each treatment arm were generated from logistic regression models adjusted for treatment arm, age, sex, day 0 parasite density and day 0 fever, with all confounders (except treatment arm) set at mean/prevalence values for all trial patients

After adjusting for treatment arm, age, sex, day 0 parasite density, and day 0 fever in separate multivariable analyses for the presence of each symptom between day 0 and day 13, substantial heterogeneity remained between study sites for the covariate-adjusted estimated proportion of patients reporting a symptom (Fig. 2), and this was also observed when restricting the analysis to symptoms occurring between day 3 to day 13 (i.e. after the resolution of acute malaria symptoms) (Fig. 3). The variation between the presence of symptoms across study sites was attenuated after adjustment for the presence of the symptom on day 0 or 1 for some symptoms (vomiting and diarrhoea) but not for others such as nausea, anorexia, abdominal pain and dizziness (Supplementary file 1). Results of a sensitivity analysis excluding patients less than 5 years were similar (Supplementary file 2).

### Variation between treatment arms

At the study site in Arba Minch, Ethiopia, anorexia between day 3 and day 13 was reported in 87.3% (62/71) of patients receiving placebo, 76.2% (112/147) of patients receiving low dose primaquine, and 83.3% (120/144) patients receiving high dose primaquine. In contrast, in Krong Pa, Vietnam, none (0/21) of the patients receiving placebo or low dose primaquine (0/43) reported anorexia compared with 7.3% (3/41) receiving high dose primaquine. In separate multivariable analyses for the presence of each symptom between day 3 and day 13, after adjusting for treatment category, age, sex, baseline parasitaemia and fever at presentation, the differences in symptom reporting were greater between study sites than between treatment arms within the same study site (Fig. 3).

### Variation in severity of symptoms across study sites

Of 1,747 patients reporting vomiting, diarrhoea, anorexia, nausea, abdominal pain or dizziness, 1,033 (59.1%) reported only mild symptoms (grade 1), 682 (39.0%) reported their most severe symptom as moderate (grade 2) and 32 (1.8%) reported their most severe symptom as severe (grade 3). For all reported symptoms, less than 2.5% of all patients reported severe symptoms across all study sites (Supplementary file 3).

## Discussion

The overall number of symptoms reported in the IMPROV study varied significantly between sites, and this difference remained apparent after adjusting for demographics, disease severity estimated by presence of fever and parasite density at enrolment, and treatment arm. One site in Ethiopia reported almost 100% of study patients suffering from anorexia compared to other sites where less than 5% patients reported anorexia. There were less extreme but still relevant differences in the reporting of other symptoms including vomiting, diarrhoea, nausea, abdominal pain and dizziness. Critically the differences in symptom reporting were greater between study sites than between treatment arms, suggesting that symptoms are best interpreted in relation to the control arm.

Differences in the circumstances under which symptoms were elicited may explain the variation between sites. Despite standardised training on how to complete the symptom questionnaire, the approach of study centres is likely to have differed in practice. At one extreme, investigators may have encouraged study participants to report every symptom, while in other sites the investigators may have been less explicit. This would be in line with previously reported differences showing that more specific questioning of study subjects resulted in more events reported compared to open-ended questioning [6]. The attitude of the study team towards symptom reporting is also likely to play a key role and is potentially shaped by cultural differences, similarly communication between study participants within their communities might have differed between sites and influenced reporting of symptoms. To further elucidate this, qualitative research would be required to better understand how a term like "anorexia" is interpreted by different people and what they mean if they describe it as mild, moderate etc.

There are several alternative explanations for these findings. Cultural differences between study sites and countries may play a role. Observed differences could be explained by variations in expression and reporting of symptoms, such as pain, between different groups of people [11–13]. However, the fact that two sites in the same country, Ethiopia, which are likely to share such cultural characteristics reported dramatically different symptom proportions, suggests that other factors are more likely to be contributing to the observed heterogeneity.

Data on self-reporting of symptoms versus reporting by parents on behalf of a paediatric patient was not available and could have contributed. However, the proportion of children under the age of 5 was overall small and a sensitivity analysis excluding patients under 5 showed similar results. The effect of schizontocidal treatments on symptoms could not be assessed due to collinearity with study site. However, results remained heterogeneous on day 3–13, following cessation of the schizontocidal treatment.

Gastro-intestinal tolerability of primaquine can be improved with food. While it was recommended to patients to take primaquine with food, patient level data on food uptake during treatment was not available and practices might have varied between sites. However, the difference in the proportion of patients reporting symptoms between the control arm and the treatment arms at each site were much smaller than between sites, suggesting that other explanations are more likely.

Finally, a large number of patients in the placebo arm reported symptoms following drug administration, this could in part be explained by symptoms attributable to malaria. In addition it seems likely that the ‘nocebo’ effect (negative consequences following the administration of a placebo) may have played a role in the perception of symptoms by some participants [14]. Researchers in some study centres may have discussed with study participants specific symptoms in greater detail during the consent process than in other centres, thus being more likely to receive an affirmative response when they check for these symptoms during follow up visits, even though the patients are in the placebo arm.

While the data collection tools for determining antimalarial efficacy have generally been well standardised [6], this has occurred to a lesser extent for safety and tolerability assessments. A survey among malaria trial investigators 10 years ago showed that a range of different methods were used to collect and record symptoms and AEs, and most trialists reported using a combination of general questioning (without reference to particular conditions or body system) and structured questions (with reference to particular conditions or body system) [15]. Such methodological differences greatly hamper the pooling of safety data in a similar way to that done for individual patient data meta-analyses focused on quantifying efficacy outcomes. These challenges have also been highlighted in a series of early trials with artemisinin-based combination therapies conducted in a single country, Uganda [16]. More recently, the WorldWide Antimalarial Resistance Network (WWARN) in collaboration with Clinical Data Interchange Standards Consortium (CDISC) developed standardised case record forms with the objective of facilitating the collection of relevant clinical data including safety and tolerability data according to Clinical Data Acquisition Standards Harmonization standards (CDASH). These data collection forms use a body system checklist including a grading matrix as well as more open-ended AE data collection forms [9]. Despite this welcome attempt at standardisation, differences in the interaction between patients and the study team are likely to continue to influence the collection of safety and tolerability data and emphasize on comparable questioning strategies should be made during training for multi-centre studies. Whether changes in study methods will result in more reliable and therefore replicable and generalisable results will be difficult, if not impossible, to demonstrate in the absence of a gold standard.

## Conclusions

The findings from our analysis suggest three pragmatic approaches to how symptom reporting in antimalarial trials could be improved. Firstly, the reporting of more severe symptoms is less skewed between sites and more likely to be reproducible compared to mild and moderate events. Trialists should therefore distinguish clearly between safety outcomes (severe symptoms) and tolerability outcomes (mild and moderate symptoms), with the latter needing to be interpreted with caution given greater heterogeneity of reporting. Secondly in multi-site studies with varied proportions of reported symptoms between sites, trialists should be clear in the reporting of the uncertainty surrounding their estimates. The range of proportions (rather than or in addition to the mean or median) may be a better way to report pooled results, reflecting the variability. Thirdly symptoms recorded in an intervention arm should be reported in relation to the control arm. A relative change in symptoms in the intervention arm compared to the control arm is much more relevant than simply recording the absolute occurrence of symptoms.

### Supplementary Information

**Additional file 1.**

## Data Availability

The data are available for access via the WorldWide Antimalarial Resistance Network (WWARN.org). Requests for access will be reviewed by a Data Access Committee to ensure that use of data protects the interests of the participants and researchers according to the terms of ethics approval and principles of equitable data sharing. Requests can be submitted via the Data Access Form available at WWARN.org/accessing-data and by email to the following address: malariaDAC@iddo.org. The WWARN is registered with the Registry of Research Data Repositories (re3data.org).

## Abbreviations

AE: Adverse event
IMPROV: Improving the radical cure of vivax malaria trial
G6PD: Glucose-6-phosphate-dehydrogenase
SOP: Standard operating procedures
IQR: Interquartile range
WWARN: WorldWide Antimalarial Resistance Network
CDISC: Clinical Data Interchange Standards Consortium
CDASH: Clinical Data Acquisition Standards Harmonization
