# Supplementary material for: The heterogeneity of symptom reporting across study sites: a secondary analysis of a randomised placebo-controlled multicentre antimalarial trial
Source: BMC Med Res Methodol. 2023 Sep 4;23:198. doi: 10.1186/s12874-023-02022-3 (PMC10476314; doi:10.1186/s12874-023-02022-3)
Supplement: Supplementary file 1 — Additional file 1. [file 12874_2023_2022_MOESM1_ESM.docx]

**Supplement: The heterogeneity of symptom reporting across study sites: A secondary analysis of a randomised placebo-controlled multicentre antimalarial trial**

**Supplementary file 1:** Covariate-adjusted estimate (95% CI) of proportion of patients reporting symptoms between day 3 and day 13 adjusting for the presence of that symptom on day 0 or 1 across study sites

**Supplementary file 2:** Sensitivity analysis excluding patients younger than 5 years of covariate-adjusted estimate (95% CI) of proportion of patients reporting symptoms between day 3 and day 13.

**Supplementary file 3:** The number and percentage of reported severe (grade 3) symptoms by site

**File 1:** Covariate-adjusted estimate (95% CI) of proportion of patients reporting symptoms between day 3 and day 13 adjusting for the presence of that symptom on day 0 or 1 across study sites


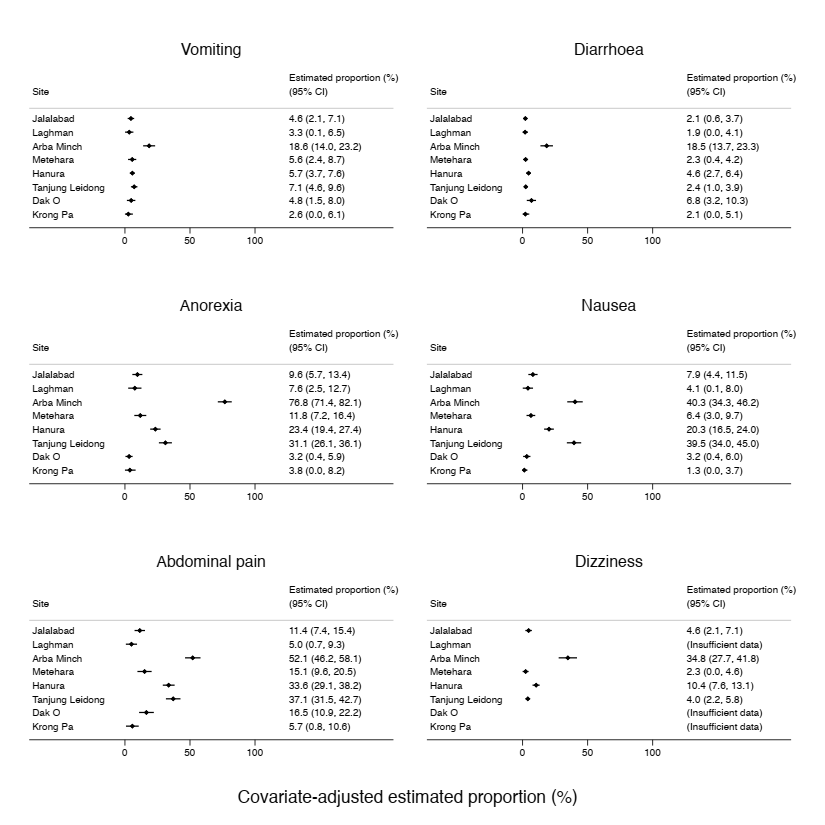


Covariate-adjusted site-specific estimated proportions were generated from logistic regression models adjusted for treatment arm, age, sex, day 0 parasite density, day 0 fever and presence of symptom on day 0 or 1, with all confounders set at mean/prevalence values for all trial patients.

**File 2:** Sensitivity analysis excluding patients younger than 5 years of covariate-adjusted estimate (95% CI) of proportion of patients reporting symptoms between day 3 and day 13.


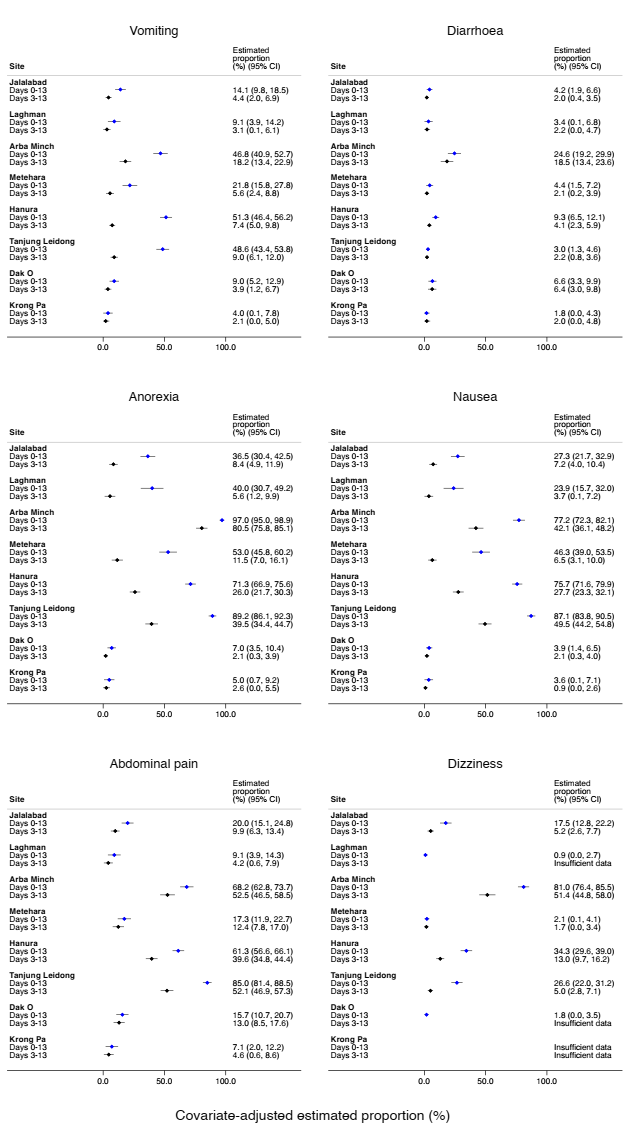


Covariate-adjusted site-specific estimated proportions were generated from logistic regression models adjusted for treatment arm, age, sex, day 0 parasite density and day 0, with all confounders set at mean/prevalence values for all included patients.

**File 3:** The number and percentage of reported severe (grade 3) symptoms by site

| **Country** | **Site** | **Patients** | **Vomiting** | **Diarrhoea** | **Anorexia** | **Nausea** | **Abdominal pain** | **Dizziness** | **Overall*** |
| --- | --- | --- | --- | --- | --- | --- | --- | --- | --- |
|  |  |  | **n (%)** | **n (%)** | **n (%)** | **n (%)** | **n (%)** | **n (%)** | **n (%)** |
| Afghanistan | Jalalabad | 311 | 1 (0.3%) | 0 (0%) | 0 (0%) | 1 (0.3%) | 2 (0.6%) | 0 (0%) | 3 (1.0%) |
|  | Laghman | 120 | 0 (0%) | 0 (0%) | 0 (0%) | 0 (0%) | 0 (0%) | 0 (0%) | 0 (0%) |
| Ethiopia | Arba Minch | 371 | 0 (0%) | 0 (0%) | 0 (0%) | 0 (0%) | 0 (0%) | 0 (0%) | 0 (0%) |
|  | Metehara | 208 | 3 (1.4%) | 0 (0%) | 1 (0.5%) | 2 (1.0%) | 0 (0%) | 0 (0%) | 4 (1.9%) |
| Indonesia | Hanura | 575 | 1 (0.2%) | 0 (0%) | 2 (3%) | 1 (0.2%) | 11 (1.9%) | 0 (0%) | 14 (2.4%) |
|  | Tanjung Leidong, | 425 | 2 (0.5%) | 1 (0.2%) | 2 (0.5%) | 5 (1.2%) | 6 (1.4%) | 0 (0%) | 9 (2.1%) |
| Vietnam | Dak O & Bu Gia Map | 219 | 1 (0.5%) | 1 (0.5%) | 0 (0%) | 0 (0%) | 1 (0.5%) | 0 (0%) | 2 (0.9%) |
|  | Krong Pa | 106 | 0 (0%) | 0 (0%) | 0 (0%) | 0 (0%) | 0 (0%) | 0 (0%) | 0 (0%) |
| **Total** |  | **2,335** | **8 (0.3%)** | **2 (0.1%)** | **5 (0.2%)** | **9 (0.4%)** | **20 (0.9%)** | **0 (0%)** | **32 (1.4%)** |

Symptom severity was defined for each individual as the maximum severity of any symptom between day 0 and day 13 (e.g. If a patient presents with the same symptom more than once during these 14 days only the presentation with the most severe symptom is recorded).

*Only includes each individual once, reporting the presence or not of a severe symptom in each individual.
